# A Novel Triboelectric Material Based on Deciduous Leaf for Energy Harvesting

**DOI:** 10.3390/mi12111314

**Published:** 2021-10-26

**Authors:** Zhuyu Ding, Ming Zou, Peng Yao, Zhiyuan Zhu, Li Fan

**Affiliations:** 1 College of Engineering and Technology, Southwest University, Chongqing 400715, China; dingzy@swu.edu.cn; 2 School of Electronic and Information Engineering, Southwest University, Chongqing 400715, China; zouming6289@email.swu.edu.cn (M.Z.); qw578592005@email.swu.edu.cn (P.Y.); zyuanzhu@swu.edu.cn (Z.Z.); 3 Ocean College, Faculty of Engineering, Zhejiang University, Zhoushan 316021, China

**Keywords:** triboelectric nanogenerator (TENG), fallen leaf, environmentally friendly materials, stacked TENG

## Abstract

Recently, the triboelectric nanogenerator (TENG) for harvesting low-frequency energy has attracted the attention of academia. However, there are few studies on environmentally friendly triboelectric materials. Here, we propose a novel triboelectric nanogenerator based on the deciduous leaf (DL-TENG) that can harvest mechanical energy from various low-frequency motions. The deciduous leaf is an environmentally friendly triboelectric material, which has a low-cost and is easy to obtain. Using it to generate electricity can achieve the effect of waste utilization. From the experimental results, the peak value of the short-circuit current (*I*_sc_) and the open-circuit voltage (*V*_oc_) can reach 4.2 µA and 150 V, respectively. The fabricated DL-TENG exhibits a stable high performance, with a maximum output power of 72.2 µW, to a load of 20 MΩ. Moreover, we also designed a stacked structure, DL-TENG, to enhance the electrical output. Additionally, the stacked DL-TENG could drive 15 commercial light-emitting diodes (LEDs). This design will promote the development of low-cost and environmentally friendly triboelectric material.

## 1. Introduction

Owing to the increasing requirements for new green energy, obtaining sustainable energy from the surrounding environment has become a hot research topic for mitigating the energy crisis. Technological innovations have unleashed the power of renewable energies such as wind, hydro, solar, and geothermal. Over the past few years, a series of energy conversion technologies, such as tidal power generators, thermoelectric generators, photoelectric generators, and wind turbine generators, have been developed [1,2,3,4]. In 2012, the triboelectric nanogenerator (TENG) was reported by the Wang group, which is based on the integration of triboelectrification and an electrostatic induction mechanism and has drawn the attention of researchers in many research fields due to its simple preparation, high electrical output, low-cost raw materials, and great application potential [5,6,7,8,9,10,11,12]. As we all know, friction motion widely exists in the environment; therefore, TENG’s acquisition of friction mechanical energy is widespread. Through a simple power generation device design and friction movement, TENG can collect almost all mechanical energy, such as wind energy, water drop energy, wave energy, human motion energy, and other mechanical energy, and convert it into electrical energy [13,14,15,16,17,18,19,20,21].

In practical applications, the electrical output of TENG devices is far lower than the theoretical output. Generally, the output performance of the TENG device will be affected by the structural design, selection of triboelectric materials, friction motion state, working environment, and other factors [22,23,24,25]. Previous studies have shown that the increase in the surfaces roughness of triboelectric materials can effectively enhance the electrical performance of TENG devices. Moreover, the difference between the gain and loss of electrons is also a relevant factor affecting the electrical output of TENG devices. Furthermore, it is worth noting that TENG devices can also act as self-powered sensors without an additional power supply equipment for various types of motion monitoring, such as vibration sensors, speed sensors, and human motion, etc. [26,27,28] So far, through the efforts of researchers, the structural design of TENG devices based on mechanical energy harvesting is becoming increasingly abundant. Additionally, many materials can be used as triboelectric materials, including organic thin-film materials, metal materials, inorganic materials, and so on [29,30,31]. However, the triboelectric materials for preparing these devices often do not consider the environmental pollution factors. In the meantime, these triboelectric materials usually require special processing equipment and professional operators to complete the production. Additionally, this restricts the low-cost and large-scale production of TENG for harvesting mechanical energy. Significantly, the triboelectric phenomenon can occur between any two materials. Thus, the TENG devices have many application prospects. Additionally, exploring new, environmentally friendly, triboelectric materials will effectively promote the development of TENG. According to previous studies [32,33], plant materials, including leaves and petals, are good triboelectric materials because they have the advantages of degradability, environmental protection, no pollution, and environmental friendliness. Therefore, developing new plant-based TENG is meaningful for the rapid development of environmentally friendly triboelectric material.

Here, we report a new deciduous leaf-based triboelectric nanogenerator (DL-TENG) that can harvest mechanical energy from various motions. Compared to previous works [32,33], DL-TENG has the advantages of a simple preparation process and a low cost, and thus is suitable for large-scale production. This way of turning fertilizer into treasure can reduce environmental pollution. Additionally, it can also simplify the preparation process, which is very beneficial in promoting the development of TENG. In this design, the polytetrafluoroethylene (PTFE) film, the deciduous leaf constitute triboelectric pair, and the aluminum foil serve as the conductive electrode. From the experimental results, the peak value of the short-circuit current (*I*_sc_) and the open-circuit voltage (*V*_oc_) could reach 4.2 µA and 150 V, respectively. Meanwhile, the transferred charge of DL-TENG could reach 38.4 nC. It is worth noting that DL-TENG has an enormous internal resistance (about 20 MΩ). Additionally, the maximum output power value of DL-TENG can reach 72.2 µW, which can power many small electronic devices. Additionally, to increase the electrical output, DL-TENG is designed as the stacked structure. The stacked DL-TENG composed of four working units can drive 15 light-emitting diodes (LEDs).

## 2. Materials and Methods

### 2.1. Preparation Process and Measurement

**Figure 1:**
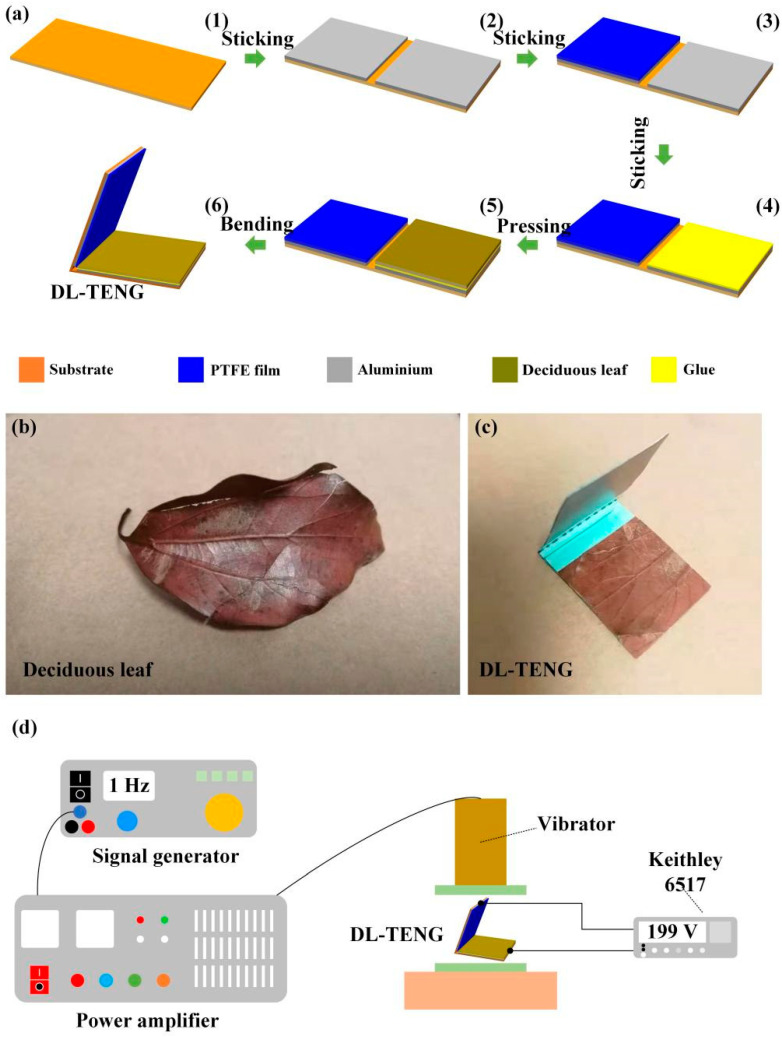
(**a**) The detailed preparation process of deciduous leaf-based triboelectric nanogenerator (DL-TENG) device. (**b**) The photograph of the deciduous leaf. (**c**) The picture of the DL-TENG. (**d**) The schematic view of the testing system.

In this work, all the used materials except deciduous leaf were commercial materials and did not need to be prepared. Figure 1 shows the detailed preparation process. The flexible polyethylene terephthalate (PET) sheet acted as the substrate due to its good material strength, as shown in Figure 1a(1). Then, two pieces of conductive aluminum foil were pasted on the substrate as the electrode of DL-TENG device, as shown in Figure 1a(2). The PTFE film was attached to aluminum foil surface, as illustrated in Figure 1a(3). Additionally, the surface of another piece of aluminum foil was covered with double-sided tape, as shown in Figure 1a(4). The deciduous leaf was cut into appropriate sizes and then pasted on the rubber surface, as shown in Figure 1a(5). Finally, the TENG device was folded into V shape to form the DL-TENG, as shown in Figure 1a(6). Figure 1b illustrates the deciduous leaf and Figure 1c shows the DL-TENG. Additionally, the thicknesses of PTFE film, deciduous leaf, PET film, and aluminum foil were 120 µm, 300 µm, 85 µm, and 110 µm, respectively. The signal generator, power amplifier, and vibrator formed the vibration system. The signal generator could control the vibration frequency, and the power amplifier could control the vibration amplitude. The electrical output (*V*_oc_, *I*_sc_, transferred charge) of DL-TENG was measured by using the Keithley 6517. The detailed test system is shown in Figure 1d.

### 2.2. Working Mechanism

**Figure 2:**
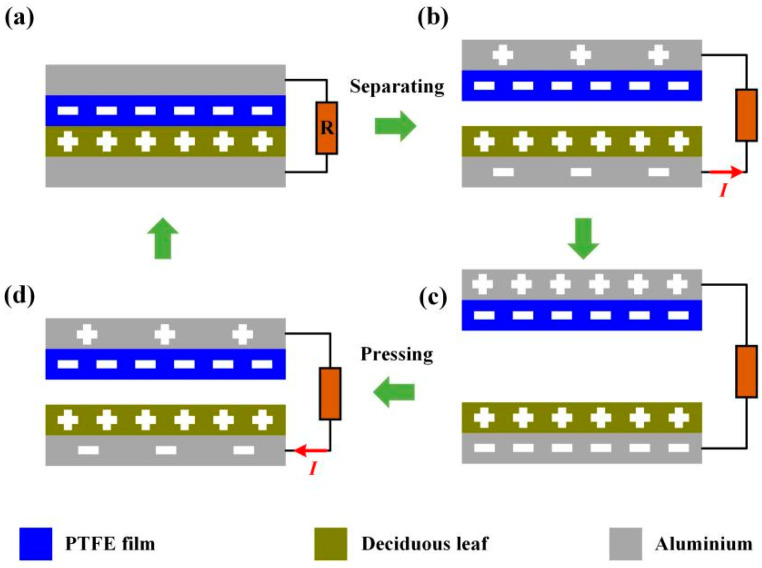
The operating principle of DL-TENG. (**a**) When the triboelectric pairs (PTFE film and deciduous leaf layer contact, positive charges and negative charges are produced on the surface of deciduous leaf layer, respectively. When the triboelectric pairs are (**a**–**c**) separating and (**c**–**a**) approaching, an internal field electrical field will be established which introduces the charges transferring between two electrodes.

The DL-TENG we proposed followed the working principle of contact-separation mode, as shown in Figure 2. According to previous work [20,21], the PTFE film has a tremendous electronic ability when it contacts other triboelectric materials. Thus, when the PTFE film surface contacts the fallen leaf surface, the electrons can transition from the deciduous leaf surface to the PTFE film surface due to the contact electrification mechanism, as shown in Figure 2a. When removing the external force contacting the PTFE film surface and the deciduous leaf surface, the surface of the PTFE film and deciduous leaf surface are separated, leading to the charge flow between the upper and lower electrodes, as shown in Figure 2b. Additionally, the moving charge produces a pulse current in the external circuit. It is important to note that the potential distribution of DL-TENG can reach equilibrium when the separation distance arrives at the maximum value, as shown in Figure 2c. When the external force is applied to the DL-TENG device again, the surfaces of the PTFE film and deciduous leaf will approach each other again and electrons near the bottom electrode will flow towards it due to the unbalanced electric field environment, as shown in Figure 2d.

Generally, the *V-Q-x* theoretical equation for the TENG device is given as:

(1) $$V=-\frac{Q}{S\varepsilon_{0}}\left(\frac{d_{1}}{\varepsilon_{1}}+\frac{d_{2}}{\varepsilon_{2}}+x\left(t\right)\right)+\frac{\sigma x\left(t\right)}{\varepsilon_{0}}$$

For the theoretical model, *d*_1_ and *d*_2_ are the thicknessed of two dielectric layers. The permittivity of two triboelectric materials and air are denoted as *ε*_1_, *ε*_2_, and *ε*_0_, respectively. *S* represents the area of two triboelectric materials, and the distance between these surfaces is set as *x*(*t*). The transferred charges between two electrodes are set as *Q*.

## 3. Results and Discussion

**Figure 3:**
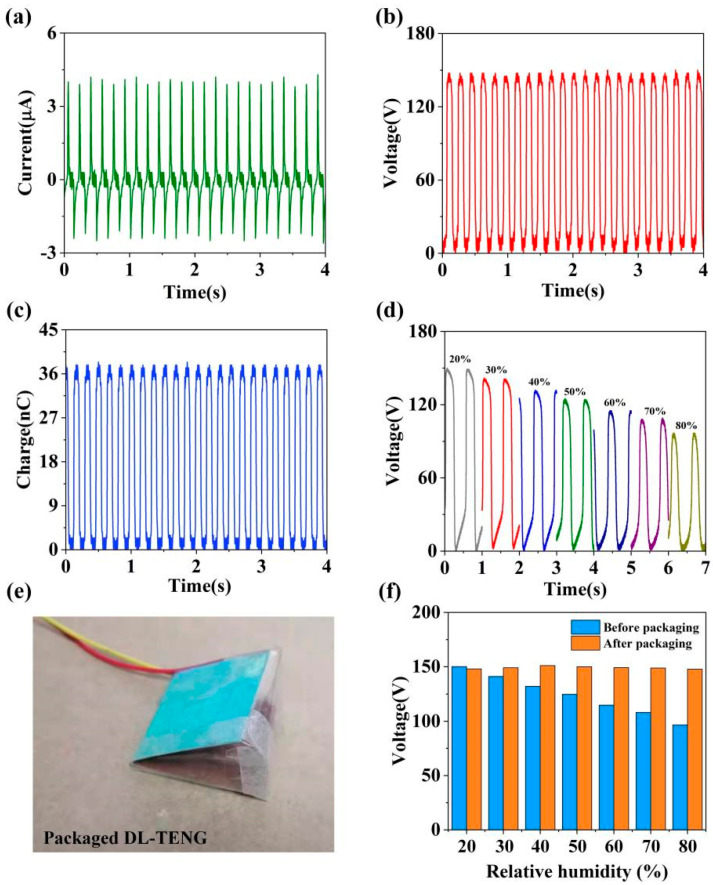
The (**a**) *I*_sc_, (**b**) *V*_oc_, and (**c**) transferred charge of DL-TENG. (**d**) The electrical output of DL-TENG under different relative humidities. (**e**) The schematic diagram of packaging structure DL-TENG. (**f**) The electrical output of DL-TENG before and after packaging under different relative humidities.

To appraise the output performance of the DL-TENG (size: 3 cm × 3 cm), we used the mechanical vibrator to provide external force for the DL-TENG working. The working frequency was set as 6 Hz, and the maximum separation distance was set as 5 mm. From the experimental results shown in Figure 3a,b, the peak value of *I*_sc_ and the *V*_oc_ reached 4.2 µA and 150 V, respectively. This characteristic, which could convert low-frequency mechanical energy into electrical energy, had potential applications in many fields, such as human motion, breeze, ocean wave energy, and so on. In the meantime, the high voltage of DL-TENG provided a good energy storage efficiency. Meanwhile, Figure 3c shows that the DL-TENG could achieve a charge transfer of 38.4 nC. Thus, the DL-TENG device had great potential in small energy storage. Moreover, considering the moisture absorption of DL-TENG, we investigated the output performance of the DL-TENG under different relative humidities, as shown in Figure 3d. According to the results, the increase in relative humidity caused a decrease in the output performance of DL-TENG because moisture on the surface of the deciduous leaf impeded electron transfer. To reduce the influence of humidity on the DL-TENG device, we designed a packaging structure, DL-TENG, as shown in Figure 3e. Figure 3f illustrates the electrical output of DL-TENG before and after packaging under different relative humidities. The results indicate that the packaging structure of DL-TENG had a good moisture resistance. Additionally, the electrical signal contained some noise, mainly because there were electromagnetic fields in the experiment, including those generated by mains power.

**Figure 4:**
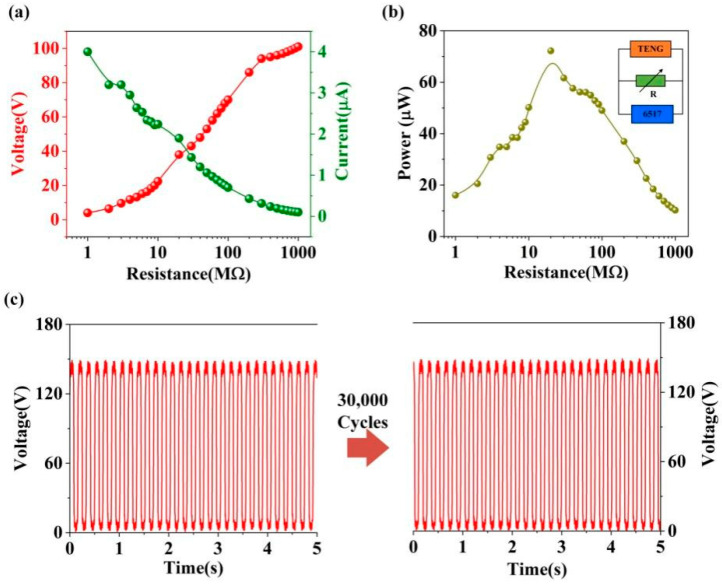
(**a**) The output voltage and output current of DL-TENG under different loads. (**b**) The output power of DL-TENG under different loads. (**c**) The reliability test of the DL-TENG.

The dependence of the electrical output (voltage and current) on a resistive load is shown in Figure 4a. The output voltage of DL-TENG increased with the increase in the external loads from 1 MΩ to 1 GΩ. Under the same conditions, the output current of DL-TENG decreased. Figure 4b shows the output power of DL-TENG under different resistances. A maximum peak power of 72.2 µW could be achieved under a resistance of 20 MΩ, corresponding to a power density of 8.02 µW/cm^2^, which was higher than the results from previous studies [34]. To test the stability of the DL-TENG device, the vibrator was set at 6 Hz to provide the periodic pressure for the DL-TENG. After continuously working for 3,000 s, the output voltage of the DL-TENG was stable, which indicated that the DL-TENG had a good stability, as shown in Figure 4c.

**Figure 5:**
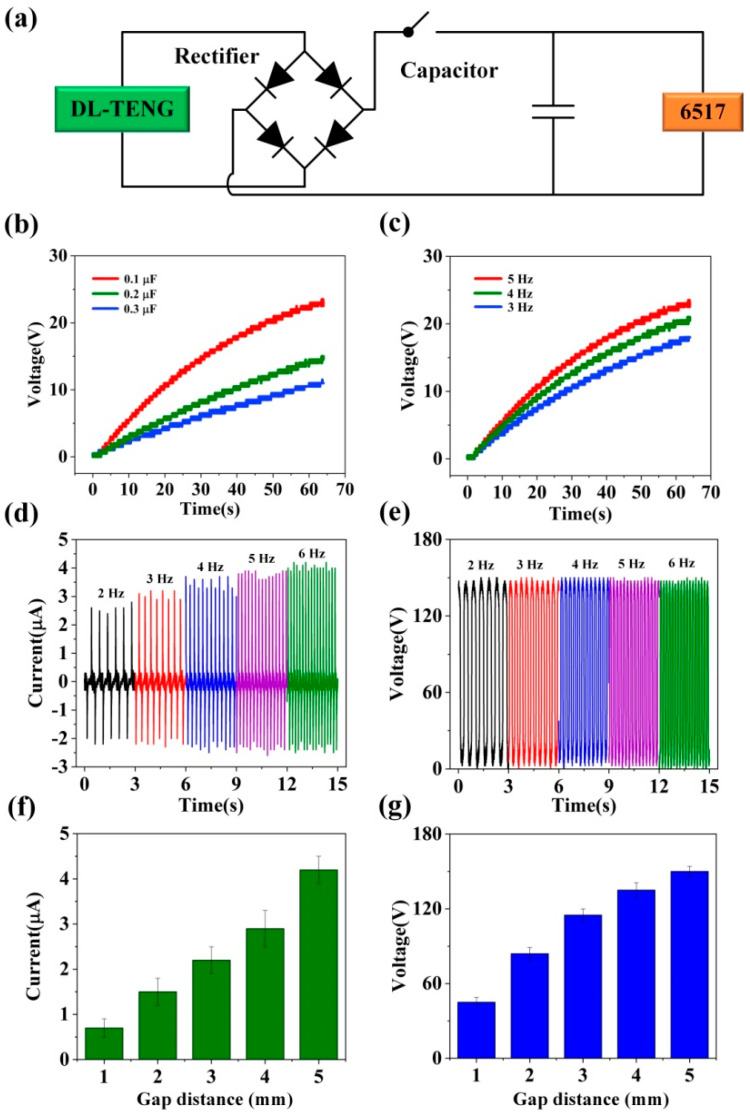
(**a**) The schematic diagram of power management circuit based on the DL-TENG. (**b**) The charging curve of DL-TENG for different capacitors under the working frequency of 5 Hz. (**c**) The charging curve of DL-TENG for 0.1 µF capacitor under different working frequencies. The (**d**) *I*_sc_ and (**e**) *V*_oc_ of DL-TENG under different working frequencies. The (**f**) *I*_sc_ and (**g**) *V*_oc_ of DL-TENG under different gap distances.

Moreover, TENGs can convert low-frequency and low-amplitude mechanical energy into electrical energy. Therefore, electrical energy storage is very important. According to previous research [3,7,12], the electrical signal was rectified by a rectifier bridge, stored in the small capacitor, and then supplied power to the electronic equipment, as shown in Figure 5a. Here, we studied the effect of DL-TENG charging capacitors at different frequencies. Obviously, the higher the externally provided vibration frequency, the faster the rate of electric energy storage, as illustrated in Figure 5b. Additionally, we analyzed the influence of DL-TENG charging different capacitors, as shown in Figure 5c. According to the experimental results, the smaller the capacitor, the faster the charging speed. Additionally, to explore the influence of operating frequency on the device, we tested the electrical performance of DL-TENG at different operating frequencies. Figure 5d shows the *I*_sc_ of DL-TENG under different working frequencies. The results showed that the *I*_sc_ of DL-TENG could grow when the working frequency increases. The reason for this result was that a high operating frequency accelerated the rate of charge transfer between the two electrodes. As illustrated in Figure 5e, when the working frequency increased, the *V*_oc_ of DL-TENG remained unchanged, which indicated that the working frequency had little effect on the *V*_oc_ of DL-TENG. Furthermore, the maximum gap distance was also an important factor affecting the performance. As shown in Figure 5f,g, when the gap distance increased, the electrical performance of DL-TENG also grew. The reason for this result was that the larger separation distance could produce a larger potential difference, which led to the improvement of electrical performance.

**Figure 6:**
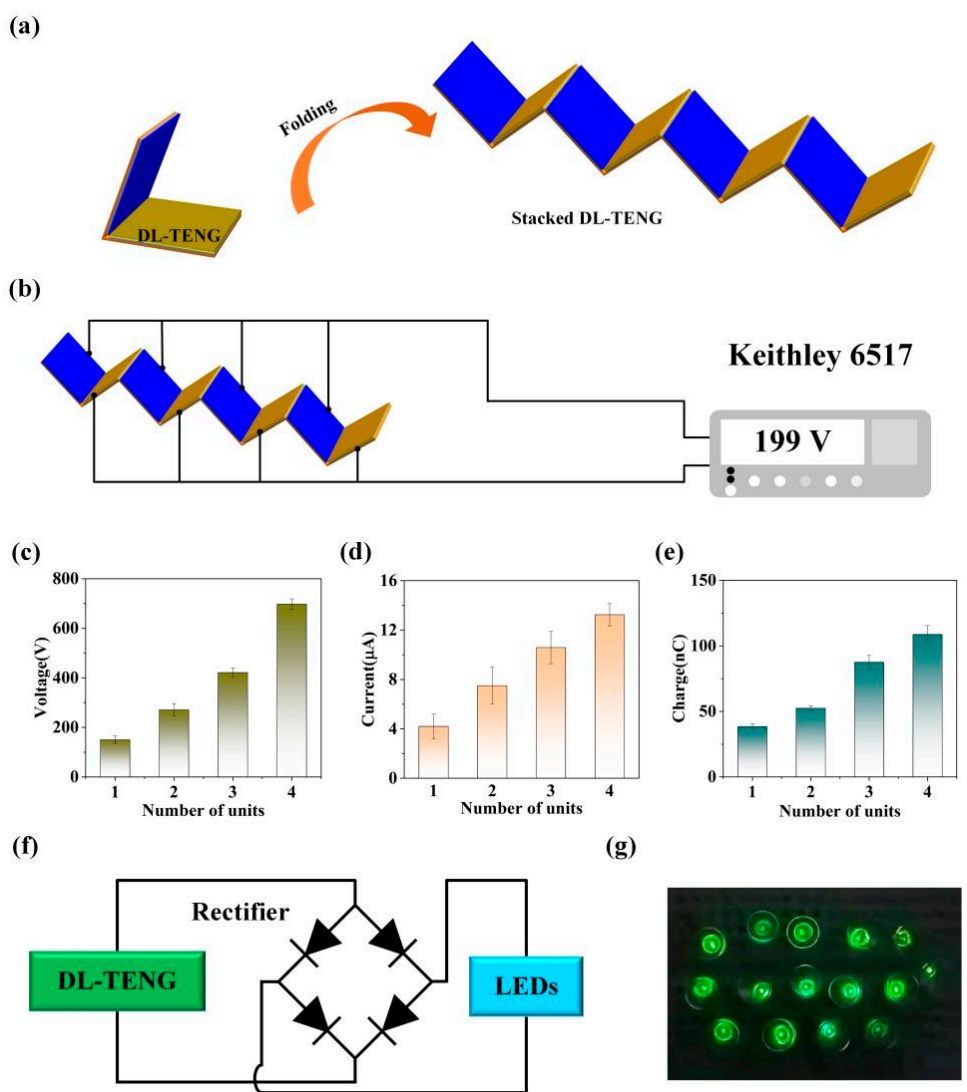
(**a**) The schematic diagram of stacked DL-TENG. (**b**) The schematic diagram of circuit connection for stacked DL-TENG. (**c**–**e**) The electrical output of DL-TENG with four working units. (**f**) The schematic diagram of power management circuit for driving commercial light-emitting diodes (LEDs). (**g**) Fifteen commercial LEDs driven by the DL-TENG device with four working units.

Additionally, to increase the electrical output, DL-TENG was designed as a stacked structure, as shown in Figure 6a. Figure 6b illustrates the circuit connection for stacked DL-TENG. Each DL-TENG unit was connected to the circuit in parallel. From the experimental results shown in Figure 6c–e, the output performance of the DL-TENG device was greatly enhanced after the stacking design. Specifically, when the number of stacking cells increased from one to four, the output voltage increased from 150 V to 698 V (Figure 6c), and this was because a parallel connection increased the contact area of the triboelectric surface. Meanwhile, the output current increased from 4.2 μA to 13.24 μA (Figure 6d) and the transfer charge increased from 38.4 nC to 108.7 nC (Figure 6e). It is worth mentioning that a parallel connection could not only increase performance but could also save the space occupied by the DL-TENG devices. The stacked DL-TENG composed of four working units could drive 15 commercial LEDs, as shown in Figure 6f,g.

## 4. Conclusions

In summary, we designed a novel deciduous leaf-based triboelectric nanogenerator (DL-TENG) that could harvest mechanical energy from low-frequency various motions. As an environmentally friendly triboelectric material, the deciduous leaf has the advantages of low cost and easy access. According to the results, the peak value of *I*_sc_ and the *V*_oc_ reached 4.2 µA and 150 V, respectively. Meanwhile, the transferred charge of DL-TENG reached 38.4 nC. A maximum peak power of 72.2 µW could be achieved under a resistance of 20 MΩ, corresponding to a power density of 8.02 µW/cm^2^, which could power many small electronic devices. To increase the electrical output, the DL-TENG was designed as the stacked structure. In future work, we will continue to explore the plant-based TENG device, continue to improve its output performance and explore new applications.

## Data Availability

Some or all data, models, or code that support the findings of this study are available from the corresponding author upon reasonable request.
